# Assessment of methylated *BCAT1* and *IKZF1* circulating tumor DNA as a prognostic biomarker in esophagogastric adenocarcinomas

**DOI:** 10.1093/dote/doag048

**Published:** 2026-05-18

**Authors:** Mei Mei Chan, Amitesh C Roy, David I Watson, Tim Bright, Lorraine Sheehan-Hennessy, Graeme P Young, Damian Hussey, Susanne K Pedersen, Jean M Winter, Erin L Symonds

**Affiliations:** Department of Oncology, Flinders Medical Centre, Southern Adelaide Local Health Network, Bedford Park, South Australia, Australia; Department of Oncology, Flinders Medical Centre, Southern Adelaide Local Health Network, Bedford Park, South Australia, Australia; Department of Surgery, Flinders Medical Centre, Southern Adelaide Local Health Network, Bedford Park, South Australia, Australia; Flinders Health and Medical Research Institute, College of Medicine and Public Health, Flinders University, Bedford Park, South Australia, Australia; Department of Surgery, Flinders Medical Centre, Southern Adelaide Local Health Network, Bedford Park, South Australia, Australia; Flinders Health and Medical Research Institute, College of Medicine and Public Health, Flinders University, Bedford Park, South Australia, Australia; Flinders Health and Medical Research Institute, College of Medicine and Public Health, Flinders University, Bedford Park, South Australia, Australia; Flinders Health and Medical Research Institute, College of Medicine and Public Health, Flinders University, Bedford Park, South Australia, Australia; Department of Surgery, Flinders Medical Centre, Southern Adelaide Local Health Network, Bedford Park, South Australia, Australia; Flinders Health and Medical Research Institute, College of Medicine and Public Health, Flinders University, Bedford Park, South Australia, Australia; Flinders Health and Medical Research Institute, College of Medicine and Public Health, Flinders University, Bedford Park, South Australia, Australia; Flinders Health and Medical Research Institute, College of Medicine and Public Health, Flinders University, Bedford Park, South Australia, Australia; Flinders Health and Medical Research Institute, College of Medicine and Public Health, Flinders University, Bedford Park, South Australia, Australia; Department of Gastroenterology, Flinders Medical Centre, Southern Adelaide Local Health Network, Bedford Park, South Australia, Australia

**Keywords:** biomarker, circulating tumor DNA, epigenetic alteration, esophagogastric cancer, risk stratify

## Abstract

Esophagogastric adenocarcinomas have poor survival, and accurate prognosis assessment is required to guide appropriate treatment. Pre-treatment planning can be challenging and would benefit from non-invasive prognostic tools. Methylated circulating tumor DNA (ctDNA) has prognostic value in colorectal cancer. The aim of the study was to evaluate the prognostic value of methylated ctDNA biomarkers in esophageal and gastric cancers.

Circulating cell free DNA, isolated from plasma collected prior to treatment from 122 patients diagnosed with esophagogastric cancer was assayed for methylated *BCAT1* and *IKZF1*. Test positivity was assessed against clinicopathological features, and prognostic factors associated with recurrence-free survival (RFS) and overall survival (OS) were investigated with Cox regression analysis.

Methylated *BCAT1/IKZF1* DNA was detected in 54.9% (67/122) of patients following a diagnosis of esophagogastric cancer. Advanced disease had higher detection rates, with a significant association between test positivity and pathological node-positive disease (odds ratio [OR] for pN2: 5.63, 95% CI 1.27–24.86, *P* = 0.02), and metastatic disease (OR 4.92, 95% CI 1.94–12.47, *P* < 0.01). A positive ctDNA result at diagnosis was associated with worse RFS for individuals considered in remission (hazard ratio [HR] 4.49, 95% CI 1.48–13.67, *P* < 0.01), and a worse OS in all patients (HR 3.64, 95% CI 1.73–7.68, *P* < 0.01), independent of stage and other clinical variables, compared to a negative ctDNA result.

Detection of circulating methylated *BCAT1*/*IKZF1* DNA is a promising prognostic biomarker in esophagogastric cancers. Prospective studies are warranted to investigate the utility of methylated *BCAT1/IKZF1* for monitoring treatment response and for risk stratification to guide adjuvant therapeutic decisions.

## INTRODUCTION

Esophageal and gastric cancers, collectively referred to as esophagogastric cancers, have poor prognosis and high mortality rate. Despite advances in treatment and some improvement in survival for esophagogastric cancers across recent decades, the 5-year survival rate for all stages combined remains below 35%.^1^ At least one-third of patients are diagnosed with metastatic disease with no opportunity for curative treatment. Esophagogastric cancer patients with metastatic disease have a median overall survival of 4–11 months.^2^^,^^3^ In patients who undergo treatment with curative intent, recurrence rates remain high at above 40%, with most occurring within 2 years of surgery.^4–6^ Accurate staging and prognostication for esophagogastric cancers, guide appropriate treatment and can improve outcomes,^7^ but reliable non-invasive biomarkers are lacking for the detection and prognosis of these cancers. Current staging and prognosis are based on assessments done with computed tomography (CT) imaging, endoscopy, endoscopic ultrasound, and positron emission tomography (PET) scanning.^8^^,^^9^ Tumor protein biomarkers used for other gastrointestinal cancers (carcinoembryonic antigen (CEA) and CA19-9) have poor sensitivity rates for esophagogastric cancers of no ˃37% and 45% for CEA and CA19-9, respectively, and no prognostic value.^10^^,^^11^

Circulating tumor DNA (ctDNA) assays show promising predictive and prognostic value in several cancers,^12–14^ including esophagogastric cancers, with the potential to guide clinical management.^15^^,^^16^ Most of the studies conducted in patients with esophagogastric cancers use tumor informed targeted mutations based on sequencing panels, with ctDNA positivity to be associated with poor prognosis and a higher rate of disease recurrence after curative resection in patients with esophagogastric adenocarcinomas.^15^^,^^17^^,^^18^ However, limitations for tumor informed approaches are costly and may result in a longer lead time for results, and that intratumor heterogeneity can mean that a DNA mutation profile informed from a biopsy is not always representative of the whole tumor.^19^ In addition, for cancers with a low tumor mutation burden, mutation-based ctDNA may be more challenging to detect.^20^ This shows a need for cost effective, ctDNA assays that are tumor agnostic and do not rely on mutational profiles.

DNA methylation is a common early event in carcinogenesis and allows the use of small, highly sensitive biomarker panels in tumor-agnostic ctDNA assays, providing greater time- and cost-efficiency than tumor-informed somatic mutation-based ctDNA assays.^21–23^ There has been limited evaluation of methylated ctDNA biomarkers for prognosis of esophagogastric adenocarcinomas at diagnosis. We have previously demonstrated the utility of using methylated biomarkers as a tumor agnostic ctDNA test in colorectal cancer. Hypermethylation of the promoter regions of branched chain amino acid transaminase 1 (*BCAT1)* and IKAROS family zinc finger 1 (*IKZF1)* DNA is 60%–78% sensitive and 92%–94% specific for the detection of primary and recurrent colorectal cancer.^24^^,^^25^ In addition, the methylated ctDNA test has been demonstrated to have prognostic value, improving clinical discrimination from staging and pathology at surgery for colorectal cancer.^26^ Given the shared endodermal origin and biological similarities of gastrointestinal adenocarcinomas, we hypothesized that detection of methylated *BCAT1* and *IKZF1* DNA may also be sensitive for the detection and prognostication of esophagogastric cancers. We further explored this using adenocarcinoma tissue cohorts from The Cancer Genome Atlas and found that both genes were differentially methylated in gastroesophageal adenocarcinomas.^27^ These findings provided the rationale to evaluate assay sensitivity in a blood based assay.

The aims of the study were to explore the positivity rates of methylated *BCAT1* and *IKZF1* DNA in esophagogastric adenocarcinomas, and to assess their pre-treatment prognostic value for prediction of recurrence-free survival (RFS) and overall survival (OS), including identification of clinicopathological factors correlating with a positive test result.

## METHODS

### Study design, participants, and setting

The analysis was conducted from a prospective study assessing methylated *BCAT1* and *IKZF1* in plasma samples collected from patients aged 18 years and above, and diagnosed with esophageal or gastric adenocarcinoma, between October 2016 and February 2021. Diagnosis was confirmed by histopathology examination of the tumor from either the primary or metastatic site. All patients newly referred to the esophago-gastric surgery unit or the medical oncology unit at Flinders Medical Centre (Adelaide, SA, Australia) were invited to participate in this study, and provided written informed consent prior to enrolment. Following written informed consent, venous blood samples were obtained before starting any treatment. A minimum of 36 months follow-up was required for RFS and OS analyses, unless cancer recurrence or death occurred earlier. Demographic information and clinical data, including cancer recurrence, cause of death, and date of death were extracted from the medical records or routine clinical care follow-up. The study protocol was approved by the Southern Adelaide Clinical Human Research Ethics Committee (reference number 162.16) and registered on the Australian New Zealand Clinical Trial Registry (reference number ACTRN12616001138471). The work conforms to the provisions of the Declaration of Helsinki.

### Clinicopathological variables

Clinical data collected pre-treatment included smoking and alcohol consumption, history of gastroesophageal reflux disease or Barrett’s esophagus, body mass index (BMI), tumor characteristics, and clinical stage (based on the AJCC Cancer Staging Eighth Edition^28^). Siewert classification were used for gastroesophageal junctional (GEJ) cancers: Type I—distal esophageal cancer (center of tumor 1–5 cm above the GEJ), type II—true carcinoma of the cardia which was further classified according to the predominant organ involved (center of cancer up to 1 cm above or 2 cm below GEJ), type III—subcardial proximal gastric cancer (center of cancer 2–5 cm below GEJ).^29^ Tumor characteristics within the esophagus or stomach included: (1) location, (2) differentiation, and (3) size at diagnosis determined as the longest diameter shown by CT/PET imaging. Treatment and surgery details were also recorded.

### Procedures and methylation testing

Blood samples were collected before the commencement of any treatment, including surgery, endoscopic resection, chemotherapy, and/or radiotherapy. Plasma was isolated by a 2-spin centrifugation (1500 g, 4°C, brakes disabled) within 4 hours of collecting blood in two 9 mL K3-EDTA tubes and placed on ice (Greiner Bio-One, Australia). Plasma was stored at −80°C and shipped to Clinical Genomics (North Ryde, NSW, Australia) for circulating cell-free analysis.

Cell-free DNA was extracted from 4.5 mL plasma using a QIASymphony Circulating Nucleic Acid Kit (Qiagen, Hilden, Germany), and bisulphite-converted using the EpiTect Fast 96 Bisulphite Conversion kit (Qiagen), with slight modifications from the manufacturer’s instructions.^30^ The bisulphite-converted DNA was analyzed for methylation of *BCAT1* and *IKZF1* by multiplex qPCR performed in triplicate on a Roche LightCycler 480 Model II instrument. Detection of *ACTB* DNA served as a positive control. Detection of methylated *BCAT1* and/or *IKZF1* within 50 cycles of PCR was classified as a positive ctDNA test result, as established in previous studies.^31^ Total methylated DNA was determined from a standard curve and expressed as a percentage of the mass of total cfDNA (*ACTB*) in plasma, as previously described.^32^ ctDNA results were not provided to the treating physicians as this was a non-interventional study.

### Statistical analysis

The sensitivity of methylated *BCAT1/IKZF1* ctDNA for the detection of esophagogastric adenocarcinomas was determined by the proportion of ctDNA positive samples, with ctDNA positivity rates assessed against cancer clinicopathological features using univariate chi-squared tests and multivariable logistic regression with odds ratios (OR) and 95% confidence intervals (CI). Methylation levels were compared between the stages of cancer with a Kruskal Wallis test with Dunn’s multiple correction test. Univariate and multivariable Cox-proportional hazards regression analysis was performed to assess the association between potential prognostic factors with RFS and OS, with hazard ratios (HR), and corresponding 95% CI reported. Variables included in the multivariable models included those where the relationship with the outcome reached a *P*-value of <0.05 in the univariate analysis, and those of clinical interest. RFS was defined as the time from the first definitive treatment (where individuals were considered in remission) to recurrence or death. OS was defined as time between the date of diagnosis and death. The RFS and OS was assessed by Kaplan–Meier analysis in the intention-to-treat population. Statistical tests were performed two-sided and a *P*-value <0.05 was considered statistically significant. All analyses were performed using STATA (version 13.1, StataCorp LCC, Texas, United States).

## RESULTS

### Patient characteristics

**Fig. 1:**
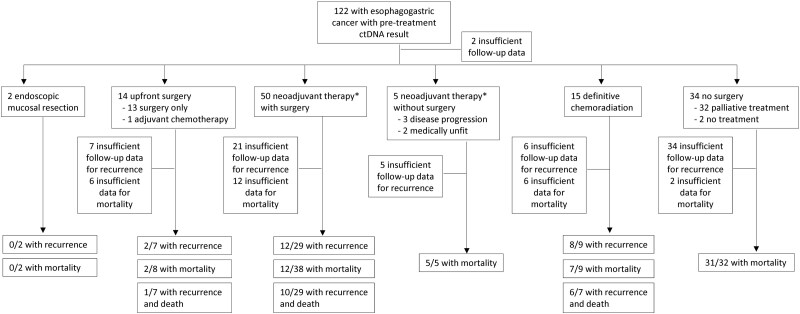
Flowchart for cohort inclusion, with recurrence and overall mortality outcomes. ^*^neoadjuvant chemoradiation or perioperative chemotherapy.

A total of 122 patients with esophagogastric adenocarcinoma were enrolled and assessed for methylated *BCAT1* and *IKZF1* ctDNA during the analyzed study period (Fig. 1). The median age of esophagogastric cancer diagnosis was 70 years (interquartile range (IQR) = 59–76) and 80.3% (98/122) were male. The primary tumor location as determined by gastroscopy included 46.7% (57/122) esophageal tumors, 31.2% (38/122) GEJ tumors, and 22.1% (27/122) gastric tumors. GEJ tumors by Siewert classification consisted of 15.8% (6/38) type I tumors, 71.0% (27/38) type II tumors, and 13.2% (5/38) type III tumors. Most patients had poorly differentiated or moderately differentiated adenocarcinomas, comprising of 36.9% and 37.7% of all cases respectively. Signet ring cell histological subtype was found in 4.1% (5/122) of patients. A large proportion of patients had advanced stage disease at presentation; 53.3% (65/122) stage III disease, and 27.9% (34/122) stage IV disease (Table 1).

**Table 1:** Baseline characteristics of patients with esophageal or gastric adenocarcinoma who were evaluated for methylated circulating tumor DNA

	**Whole cohort (%)**	**Esophageal (%)**	**Gastric (%)**
Sample size	122 (100%)	81 (66.4%)	41 (33.6%)
Age (years) – median (IQR^a^)	70.0 (58.8–76.0)	70.0 (59.5–76.0)	69.0 (55.0–78.0)
**Sex**			
Male	98 (80.3%)	68 (83.9%)	30 (73.2%)
Female	24 (19.7%)	13 (16.1%)	11 (26.8%)
**Primary tumor location**			
Esophagus	57 (46.7%)		
* Upper*	1 (0.8%)	1 (1.2%)	
* Mid*	5 (4.1%)	5 (6.2%)	
* Lower*	51 (41.8%)	51 (63.0%)	
Gastroesophageal junction	38 (31.2%)	24 (29.6%)	14 (34.1%)
Stomach	27 (22.1%)		
* Cardia/body/lesser and greater curvature*	17 (13.9%)		17 (41.5%)
* Incisura/antrum/pylorus*	8 (6.6%)		8 (19.5%)
* Diffuse (Linitis plastica)*	2 (1.6%)		2 (4.9%)
**Siewert classification for GOJ tumors**			
Siewert type 1	6 (15.8%)	6 (7.4%)	0
Siewert type 2 Siewert type 3	27 (71.0%) 5 (13.2%)	18 (22.2%) 0	9 (21.9%) 5 (12.2%)
**Differentiation grade**			
Well differentiated	9 (7.4%)	8 (9.9%)	1 (2.4%)
Moderately differentiated	45 (36.9%)	35 (43.2%)	10 (24.4%)
Poorly differentiated	46 (37.7%)	26 (32.1%)	20 (48.8%)
Signet ring cells	5 (4.1%)	0	5 (12.2%)
Unknown	17 (13.9%)	12 (14.8%)	5 (12.2%)
**AJCC cancer staging at diagnosis (clinical)**			
Stage I	8 (6.6%)	5 (6.2%)	3 (7.3%)
Stage II	15 (12.3%)	11 (13.6%)	4 (9.8%)
Stage III	65 (53.3%)	43 (53.1%)	22 (53.7%)
Stage IV	34 (27.9%)	22 (27.2%)	12 (29.3%)
**Treatment intent**			
Curative	85 (69.7%)	57 (70.4%)	28 (68.3%)
Palliative	34 (27.9%)	22 (27.2%)	12 (29.3%)
No treatment	1 (0.8%)	0	1 (2.4%)
Unknown	2 (1.6%)	2 (2.5%)	0
**Smoking status**	(n = 115)	(n = 74)	(n = 41)
Current	20 (17.4%)	12 (16.2%)	8 (19.5%)
Previous	56 (48.7%)	41 (55.4%)	15 (36.6%)
Never	39 (33.9%)	21 (28.4%)	18 (43.9%)
**Alcohol intake status**	(n = 117)	(n = 77)	(n = 40)
Light to none	66 (56.4%)	37 (48.1%)	29 (72.5%)
Moderate	40 (34.2%)	31 (40.3%)	9 (22.5%)
Heavy^a^	11 (9.4%)	9 (11.7%)	2 (5.0%)
**Esophageal conditions**			
Gastroesophageal reflux disease	38 (31.2%)	27 (33.3%)	11 (26.8%)
Barrett’s esophagus	34 (27.9%)	32 (39.5%)	2 (4.9%)
**Body mass index (kg/m** ^**2**^**)**	(n = 115)	(n = 77)	(n = 38)
<25	41 (35.65%)	20 (26.0%)	21 (55.3%)
25–29.9	41 (35.65%)	31 (40.3%)	10 (26.3%)
≥30	38 (28.7%)	26 (33.8%)	7 (18.4%)
**ctDNA detected**			
Yes	67 (54.9%)	45 (55.6%)	22 (53.7%)
No	55 (45.1%)	36 (44.4%)	19 (46.3%)

ctDNA: circulating tumor DNA; GOJ: gastroesophageal junction; IQR: interquartile range.

a Heavy considered >4 standard drinks/day.

Eighty-five (85/122, 69.7%) patients were offered curative intent treatment (treatment was not offered when disease was advanced and non-operable, or when comorbidities precluded treatment). Of these 85 patients, 62.3% (53/85) underwent neoadjuvant therapy involving either chemoradiation or perioperative chemotherapy before planned surgery (gastrectomy or esophagectomy). Fifteen of the 85 patients (17.6%) underwent definitive chemoradiation for unresectable esophagogastric cancer, 14 (16.5%) had upfront surgery, and 2 (2.4%) underwent endoscopic mucosal resection for early stage disease. One patient (1.2%) with oligometastatic disease received definitive chemoradiation to the primary and stereotactic ablative body radiation therapy to the oligometastatic site. Among those who underwent neoadjuvant therapy, 3 were upstaged to stage IV disease at surgery, and 2 were subsequently deemed too frail for surgery due to treatment-related toxicities and other complicating comorbidities. Table 1 summarizes patient characteristics.

### Detection of methylated ctDNA biomarkers and association with clinicopathological features

**Fig. 2:**
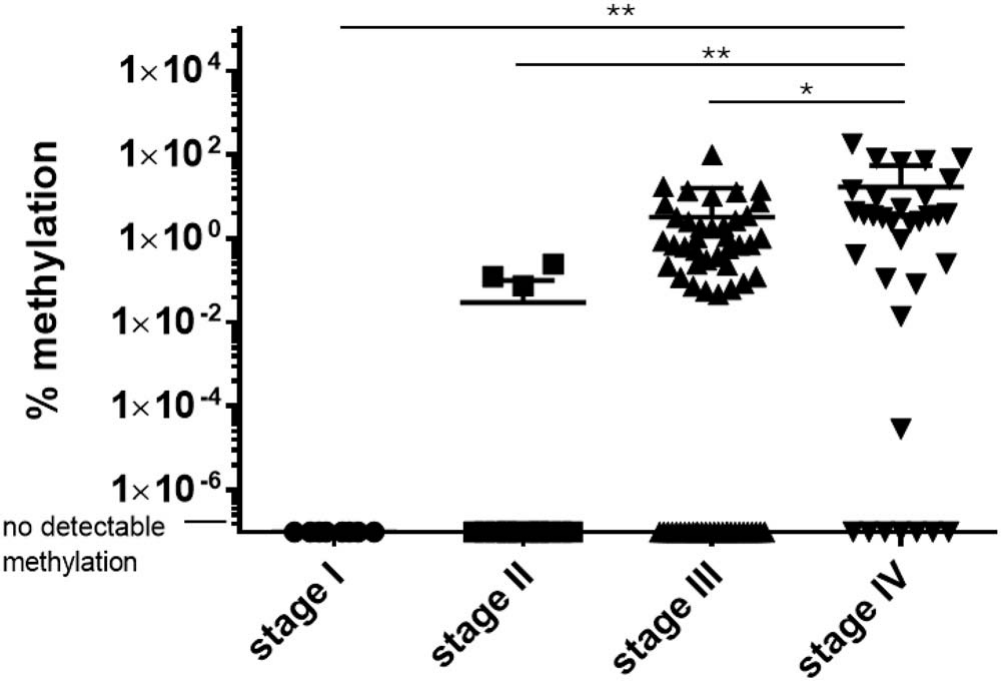
Individual levels of methylation of circulating tumor DNA for different stages of esophageal and gastric cancers: stage I (n = 8), stage II (n = 15), stage III (n = 65), stage IV (n = 34). ^*^P < 0.05, ^**^P < 0.01.

The positivity rate (sensitivity) of methylated ctDNA for esophagogastric cancers pre-treatment was 54.9% (67/122). There was no difference between the ctDNA sensitivity for esophageal and gastric cancers (55.6% [45/81] vs 53.7% [22/41], respectively, *P* = 0.842). When considering the AJCC stage of cancer, no stage I cancers were detected (0/8), 20% of stage II were detected (3/12), while sensitivity was 56.9% for stage III (37/65) and 79.4% for stage IV (27/34). Methylation levels were associated with stage of cancer, with the highest levels observed in stage IV (Fig. 2, *P* < 0.05).

In univariate analysis (Table 2), there was a significant association between a positive ctDNA result and pathological node-positive disease (pN2: OR 5.63, 95%CI 1.27–24.86, *P* = 0.02, pN3: OR 11.25, 95% CI 1.03–123.24, *P* = 0.048), tumor size at diagnosis (OR 1.03, 95% CI 1.01–1.05, *P* = 0.01) and presence of metastatic disease (OR 4.92, 95% CI 1.94–12.47, *P* < 0.01). When considering AJCC stage instead of separately considering node involvement and metastatic disease, compared to stage II, there was a non-significant increase in odds ratio for the association between stage III and a positive ctDNA test (OR 5.29, 95% CI 1.36–20.53, *P* = 0.02) and a significant association with stage IV disease (OR 15.43, 95% CI 3.40–70.11, *P* < 0.01). Stage of disease remained significantly associated with ctDNA positivity following adjustment for age, sex, and tumor size (Stage IV: OR 7.19, 95% CI 1.29–40.07, *P* = 0.02).

**Table 2:** Univariate analysis of demographic and clinicopathological variables assessed against ctDNA positivity

**Variable (sample size)**	**Unadjusted odds ratio (95% CI)**	***P*-value**	**Adjusted odds ratio (95% CI)**	***P*-value**
Age (n = 122)	1.00 (0.97–1.03)	0.82	0.99 (0.95–1.03)	0.68
Sex (n = 122; reference female)				
Male	1.58 (0.64–3.86)	0.32	1.66 (0.48–5.68)	0.42
BMI (n = 115; reference <25 kg/m^2^)				
25–29 kg/m^2^	0.67 (0.28–1.62)	0.37	–	–
≥30 kg/m^2^	0.61 (0.24–1.56)	0.30	–	–
Smoking status (n = 115; reference non-smoker)				
Ex-smoker	1.03 (0.45–2.35)	0.94	–	–
Active smoker	0.77 (0.26–2.28)	0.64	–	–
Drinking (n = 117; reference none to light)				
Moderate	0.92 (0.42–2.02)	0.84	–	–
Heavy	2.22 (0.54–9.13)	0.27	–	–
Site of primary (n = 122; reference esophageal)				
GOJ	0.68 (0.30–1.55)	0.35	–	–
Gastric	0.73 (0.29–1.83)	0.50	–	–
Polypharmacy (n = 119)	1.49 (0.65–3.41)	0.35	–	–
Histological (n = 105; reference moderately differentiated^a^)				
Poorly differentiated	1.04 (0.45–2.38)	0.93	–	–
Well differentiated	0.23 (0.04–1.22)	0.09	–	–
Signet ring cells	0.53 (0.08–3.51)	0.51	–	–
Lymphovascular invasion (n = 59)	0.69 (0.22–2.17)	0.52	–	–
Perineural invasion (n = 58)	1.45 (0.42–4.95)	0.55	–	–
pN-stage (n = 64; reference pN0 disease)				
pN1 disease	3.75 (0.95–14.82)	0.06	^	^
pN2 disease	5.63 (1.27–24.86)	0.02	^	^
pN3 disease	11.25 (1.03–123.24)	0.048	^	^
Presence of metastatic disease (n = 122)	4.92 (1.94–12.47)	<0.01	^	^
AJCC stage (n = 122; reference = stage II)				
Stage I	N/A	N/A	N/A	N/A
Stage III	5.29 (1.36–20.53)	0.02	2.94 (0.67–12.94)	0.15
Stage IV	15.43 (3.40–70.11)	<0.01	7.19 (1.29–40.07)	0.02
Tumor size at diagnosis (n = 98; per mm)	1.03 (1.01–1.05)	<0.01	1.01 (0.99–1.03)	0.68

a Selected as the reference as a clinically relevant intermediate category between well- and poorly differentiated tumors.

^Excluded due to collinearity with AJCC stage.

BMI: body mass index; CI: confidence interval.

### Disease recurrence and survival analysis

Assessment of recurrence was conducted in 47 individuals. These were individuals who had curative intent treatment and were considered in remission, and who had at least 36 months follow-up, unless recurrence occurred earlier. This analysis excluded five patients who underwent curative intent treatment but did not complete the scheduled course or did not proceed to surgery due to progressive disease. The median follow-up from treatment commencing was 49.3 months (IQR 42.9–59.2 months) for patients who did not develop recurrence. Of the 47 patients analyzed, 46.8% of patients (22/47) developed disease recurrence, including 1 stage I, 2 stage II, and 19 stage III at initial diagnosis. The median time to recurrence was 10.2 months (IQR 7.3–17.3 months) after the start of treatment. Patients who underwent surgery had a 63% lower risk for developing recurrence (HR 0.37, 95% CI 0.15–0.90, *P* = 0.03) compared to those who did not have surgery. There was a higher risk of recurrence following R1/R2 resection compared with those with R0 resection (HR 6.42, 95% CI 1.79–23.03, *P* < 0.01). Individuals with pN3 disease also had a higher risk of recurrence compared to pN0 disease (HR 5.50, 95% CI 1.03–29.49, *P* = 0.046).

**Fig. 3:**
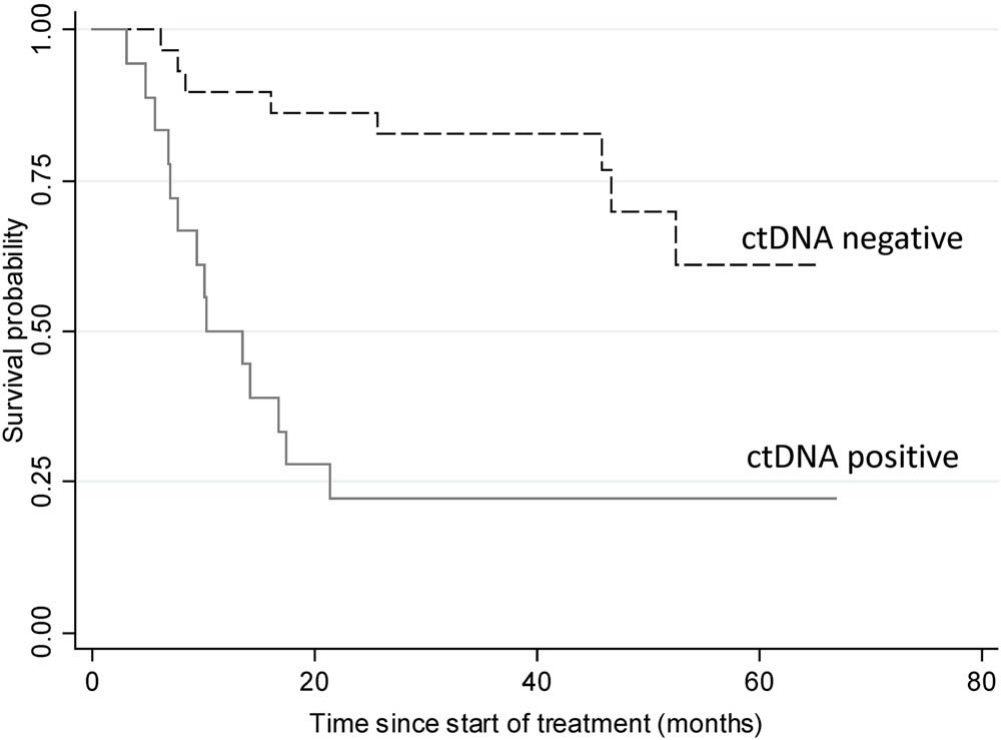
Recurrence-free survival Kaplan–Meier analysis comparing patients with or without detectable circulating tumor DNA (ctDNA).

Of the 22 individuals who developed disease recurrence, 63.6% (14/22) had a positive ctDNA result at diagnosis, including 1 stage II and 13 stage III, one of which was later upstaged to stage IV. A ctDNA positive result at diagnosis had a HR of 4.71 (95% CI 1.95–11.37, *P* < 0.01) for recurrence compared to those with a negative ctDNA (Fig. 3). Following adjustment with multivariable analysis, a positive ctDNA result was significantly associated with disease recurrence (HR 4.69, 95% CI 1.58–13.95, *P* < 0.01), independent of age, sex, BMI, location of cancer, completion of surgery, and AJCC stage (Table 3).

**Table 3:** Cox proportional hazards regression analysis of factors associated with recurrence-free survival

**Variable**	**Unadjusted HR (95% CI)**	***P*-value**	**Adjusted HR (95% CI)**	***P*-value**
Age (n = 47)	1.01 (0.97–1.05)	0.67	1.00 (0.96–1.05)	0.91
Sex (n = 47; reference female) Male	0.88 (0.32–2.40)	0.81	0.66 (0.18–2.49)	0.54
Site of primary (n = 47; reference esophageal)				
Gastric	0.36 (0.13–0.99)	0.05	0.38 (0.10–1.45)	0.16
Surgery (n = 47; reference no surgery)	0.37 (0.15–0.89)	0.03	0.65 (0.22–1.95)	0.44
BMI ≥30 (n = 44; reference <25 kg/m^2^)				
BMI 25–29.9	0.84 (0.33–2.13)	0.72	0.33 (0.09–1.22)	0.10
BMI ≥30	0.25 (0.07–0.93)	0.04	0.23 (0.06–0.94)	0.04
Positive pre-treatment ctDNA (n = 47; reference negative ctDNA)	4.71 (1.95–11.37)	<0.01	4.69 (1.58–13.95)	0.01
pN-stage (n = 36; reference pN0 disease)				
pN1 disease	1.43 (0.34–6.01)	0.62	^	^
pN2 disease	2.78 (0.74–10.43)	0.13	^	^
pN3 disease	5.50 (1.03–29.49)	0.046	^	^
Metastatic disease (n = 47; reference no metastatic disease)	3.67 (0.47–28.40)	0.21	–	–
AJCC Stage (n = 47; reference stage II)				
Stage I	0.63 (0.06–6.94)	0.70	1.13 (0.06–19.85)	0.93
Stage III	2.16 (0.50–9.28)	0.30	1.35 (0.24–7.77)	0.73
Stage IV	N/A	N/A	N/A	N/A
R1/R2 resection (n = 30; reference R0)	6.42 (1.79–23.03)	<0.01	**-**	**-**
Alcohol consumption (n = 47; reference light to none)				
Moderate	0.65 (0.25–1.71)	0.39	**-**	**-**
Heavy (> 4 standard drinks/day)	3.33 (0.72–15.33)	0.12	**-**	**-**

^Excluded due to collinearity with AJCC stage.

BMI: body mass index; CI: confidence interval; ctDNA: circulating tumor DNA; HR: hazard ratio.

**Fig. 4:**
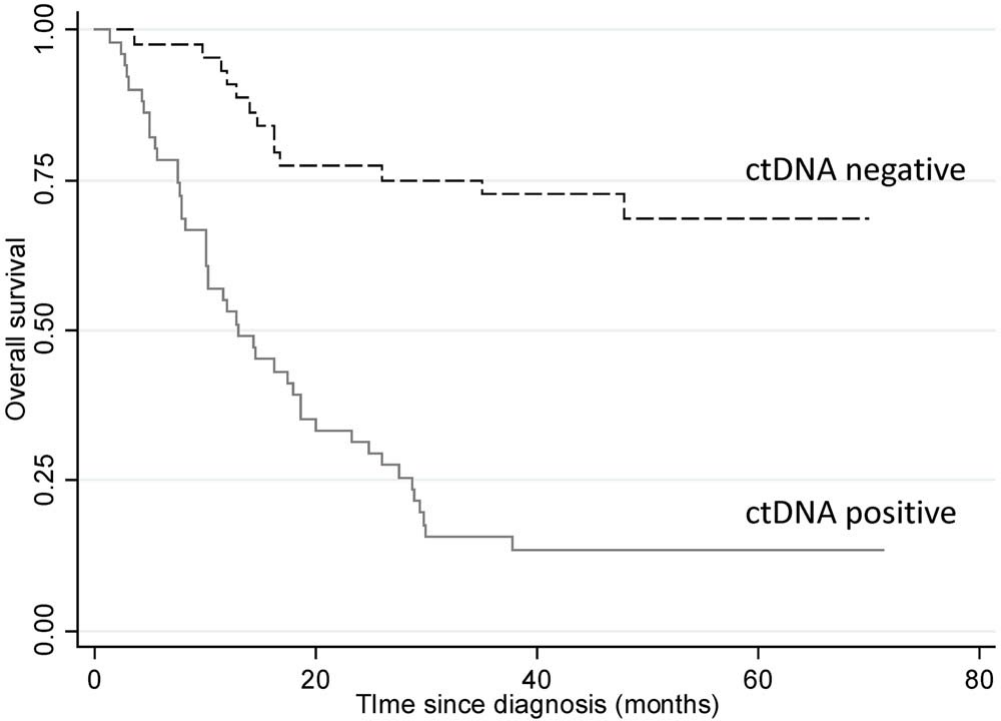
All-cause mortality Kaplan–Meier analysis comparing patients with or without detectable circulating tumor DNA (ctDNA).

Overall survival was assessed in 95 individuals who were followed for 36 months or until death from any cause, regardless of treatment received. The median overall survival for the cohort was 26.1 months (IQR 10.9–46.2 months). There were 57 deaths, of which 77.2% (44/57) had a positive ctDNA at diagnosis. All-cause mortality was more than five times higher in patients testing positive for ctDNA (HR 5.43, 95% CI 2.89–10.18, *P* < 0.01) compared to those testing negative in a univariate analysis (Fig. 4). Other factors associated with all-cause mortality are summarized in Table 4. There were 46 deaths that were confirmed to be due to the cancer. Cancer-specific mortality analysis yielded similar results for patients with a positive ctDNA, with a hazard ratio of 5.44 (95% CI 2.74–10.79, *P* < 0.001). Multivariable analysis showed that a positive pre-treatment ctDNA remained a predictor of overall survival (HR 3.64, 95% CI 1.73–7.68, *P* < 0.01), independent of age, sex, BMI, alcohol intake, location of cancer, surgery, and AJCC stage.

**Table 4:** Cox proportional hazards regression analysis of factors associated with all-cause mortality

**Variable**	**Unadjusted HR (95% CI)**	***P*-value**	**Adjusted HR (95% CI)**	***P*-value**
Age (n = 95)	1.02 (0.99–1.04)	0.16	1.02 (0.99–1.05)	0.19
Sex (n = 95; reference female)				
Male	1.01 (0.52–1.95)	0.98	0.65 (0.28–1.46)	0.30
Site of primary (n = 95; reference esophageal)				
Gastric	0.77 (0.44–1.34)	0.35	1.00 (0.50–1.98)	0.99
Surgery (n = 95; reference no surgery)	0.20 (0.11–0.36)	<0.01	0.52 (0.21–1.28)	0.15
BMI ≥30 (n = 89; reference <25 kg/m^2^)				
BMI 25–29.9	0.74 (0.42–1.32)	0.31	0.54 (0.27–1.09)	0.09
BMI ≥30	0.40 (0.19–0.85)	0.02	0.56 (0.25–1.23)	0.15
Positive pre-treatment ctDNA (n = 95; reference negative ctDNA)	5.43 (2.89–10.18)	<0.01	3.64 (1.73–7.68)	<0.01
pN-stage (n = 46; reference pN0 disease)				
pN1 disease	1.88 (0.45–7.89)	0.39	^	^
pN2 disease	3.75 (1.01–13.99)	0.049	^	^
pN3 disease	5.34 (1.02–27.96)	0.047	^	^
Metastatic disease (n = 95; reference no metastatic disease)	4.79 (2.75–8.35)	<0.01	^	^
AJCC Stage (n = 95; reference stage II)				
Stage I	N/A	N/A	N/A	N/A
Stage III	2.36 (0.71–7.82)	0.16	0.66 (0.17–2.53)	0.54
Stage IV	8.80 (2.63–29.38)	<0.01	1.51 (0.34–6.61)	0.59
R1/R2 resection (n = 39; reference R0)	5.47 (1.77–16.94)	<0.01	–	–
Alcohol consumption (n = 91; reference light to none)				
Moderate	0.78 (0.43–1.42)	0.42	0.79 (0.40–1.55)	0.49
Heavy (> 4 standard drinks/day)	2.52 (1.14–5.54)	0.02	1.87 (0.77–4.54)	0.17

^Excluded due to collinearity with AJCC stage.

BMI: body mass index; CI: confidence interval; ctDNA: circulating tumor DNA; HR: hazard ratio.

## DISCUSSION

This study has shown utility for methylated *BCAT1* and *IKZF1* as ctDNA biomarkers for prognosis of esophagogastric cancers. The overall sensitivity of the methylated *BCAT1* and *IKZF1* ctDNA test in detecting esophagogastric adenocarcinomas was 55%, with higher detection rates correlating with more advanced disease stages. However, use of these methylation markers as population screening tests may be limited, given the low sensitivity for detection of early stage disease seen here in the small number of early stage cases that were available. Our study findings are similar to the findings of recent studies of mutation-based ctDNA in esophagogastric cancers, supporting the feasibility and prognostic value of methylated *BCAT1* and *IKZF1* ctDNA in both localized and metastatic disease.^33^^,^^34^

The ctDNA markers were equally sensitive for esophageal (55.6%) and gastric cancers (53.7%). In comparison with findings from a meta-analysis, the pooled sensitivity using targeted sequencing methodologies for detection of esophageal cancer was 71% (range 55.7%–82.6%),^16^ however this review combined esophageal adenocarcinomas and squamous cell carcinomas in their analysis. Many of the studies reviewed used tumor-informed next-generation sequencing, including somatic alterations of *TP53*, *PIK3CA*, *ERBB2*, and *KRAS* which are common in esophagogastric cancers.^15^^,^^35^ In our study, there were higher rates of positive ctDNA in patients with more advanced stage disease, particularly stage III and stage IV (57% and 79%, respectively). Similarly, a study assessing a multicancer targeted methylation-based ctDNA blood test for the early detection of multiple cancers showed an improved sensitivity with increasing stage for esophagogastric cancers.^36^ Detection rates were significantly lower in patients with stage I esophageal and gastric cancers (12.5% and 16.7%, respectively) in comparison to other cancer types, with sensitivity rates ranging from 21.9% to 100%.^36^ Mutation based ctDNA detection also has lower sensitivity rates in early stage disease, with a study in gastric cancer that used an informed targeted sequencing panel including 1021 genes reporting that no patients with early stage gastric adenocarcinoma had detectable ctDNA, while 68% of stage III cases were ctDNA positive.^37^ None of the patients with stage I disease had a positive ctDNA in the current study. A plausible explanation for this is that early stage luminal gastrointestinal cancers are less likely to shed ctDNA into the bloodstream,^35^^,^^38^ but the limitation for this analysis is the small sample size for stage I esophagogastric cancers (n = 8). Larger studies are required to validate the sensitivity and specificity of the methylated markers studied herein. Although the sensitivity of *BCAT1* and *IKZF1* ctDNA markers were lower than observed in other studies using tumor-informed ctDNA, which involves a larger panel of targeted gene sequencing, it is a simple non-invasive, cost-efficient agnostic test with a shorter turnaround time.

Our study supports the feasibility and prognostic value of the two methylated ctDNA markers in esophagogastric cancers. We observed that a positive pre-treatment ctDNA is highly prognostic of poorer patient outcomes, with a significantly higher risk of recurrence after curative therapy (HR 4.69) and a poorer survival (HR 3.64) by multivariable analysis, independent of stage of cancer. A high proportion of patients who developed recurrence or died had detectable ctDNA at diagnosis (63.6% and 77.2%, respectively), an observation consistent with previous reports using different ctDNA assays.^17^^,^^35^^,^^39^^,^^40^ This suggests that the presence of ctDNA is an indicator for a poor prognosis and may indicate a more aggressive cell turnover resulting in ctDNA shedding. It is well understood that tumor ctDNA shedding increases with tumor burden, nodal status, histology, and the mitotic activity of the tumor.^41–43^ In our study, factors that were significantly associated with a positive ctDNA included node-positive disease and metastatic disease. A higher pN-stage was significantly associated with a higher risk of recurrence on univariate analysis. However, patients with accurate nodal assessment were limited only to those who underwent surgery, resulting in a smaller sample size, and this association is likely a reflection of more advanced cancer. The association of ctDNA positivity with clinical measures of poorer prognosis supports future studies for using ctDNA to guide treatment and surveillance strategies for esophagogastric adenocarcinomas.

Given the potential prognostic utility of the methylation markers in the pre-treatment setting, further studies are also warranted to assess the potential of ctDNA analysis in detection of minimal residual disease post-curative intent treatment. Several studies of esophagogastric cancers have found that patients with detectable ctDNA at any post-treatment time point had a higher risk of recurrence and a worse prognosis,^44–46^ with molecular relapse generally preceding clinical relapse by several months.^37^^,^^47^ In the longitudinal PLAGAST trial it was found that persistent tumor-informed ctDNA positivity was associated with poor outcomes in 62 patients with gastric and GEJ adenocarcinomas.^48^ In addition, in the phase 2 PANDA trial, presurgical tumor-informed ctDNA results were associated with pathologic response,^49^ but sample size was limited (n = 20). Future studies of longitudinal ctDNA monitoring, particularly in the absence of tumor sequencing, could be used to identify patients at risk of recurrence, as well as selecting patients that could benefit from treatment escalation in esophagogastric cancers.

Limitations of this observational study include the modest sample size, variation in perioperative or neoadjuvant treatment regimens, and other unadjusted confounding factors, which may cause an overestimate of the association. It must also be noted that the biomarkers explored do not have FDA or other regulatory approvals and therefore have been used in an observational setting. A further limitation is that while the sensitivity of the methylated *BCAT1/IKZF1* ctDNA was similar to that reported in studies using mutation-based ctDNA,^50^ the current study did not perform a direct comparison. The strength of our study is the long follow-up time (median follow-up of 49.3 months), and it is one of the first studies to assess the use of methylated ctDNA for prognosis assessment in esophageal, GEJ and gastric adenocarcinomas.

## CONCLUSION

In conclusion, this exploratory study has shown that *BCAT1* and *IKZF1* methylated ctDNA biomarkers have potential prognostic significance in esophagogastric cancers. These methylation markers have a good sensitivity in advanced stage esophagogastric cancers. A positive ctDNA result at diagnosis was an independent prognostic biomarker of survival. These findings provide proof of concept that ctDNA methylation of *BCAT1 and IKZF1* can be detected in the plasma of patient with esophagogastric adenocarcinomas. Future research should assess the role of methylated ctDNA *BCAT1*/*IKZF1* for the monitoring of treatment response in advanced stage esophagogastric cancers, including its potential for use alongside tumor-informed mutation testing, as this approach may improve sensitivity compared with assays that rely only on mutation profiles.

## Data statement

The data that support the findings of this study are available from the corresponding author upon reasonable request.

## Notes

Specific author contributions: Mei Mei Chan (Conceptualization, Data curation, Formal analysis, Investigation, Methodology, Visualization, Writing—original draft, Writing—review & editing), Amitesh Roy (Conceptualization, Funding acquisition, Investigation, Resources, Supervision, Writing—review & editing), David Watson (Conceptualization, Funding acquisition, Investigation, Methodology, Project administration, Resources, Supervision, Writing—review & editing), Tim Bright (Project administration, Resources, Supervision, Writing—review & editing), Lorraine Sheehan-Hennessy (Investigation, Project administration, Resources, Writing—review & editing), Graeme P. Young (Conceptualization, Funding acquisition, Methodology, Writing—review & editing), Damian Hussey (Conceptualization, Funding acquisition, Resources, Writing—review & editing), Susanne K. Pedersen (Conceptualization, Funding acquisition, Investigation, Methodology, Resources, Validation, Writing—review & editing), Jean Winter (Data curation, Formal analysis, Funding acquisition, Investigation, Methodology, Project administration, Writing—review & editing), and Erin Leigh Symonds (Conceptualization, Data curation, Formal analysis, Funding acquisition, Investigation, Methodology, Project administration, Supervision, Visualization, Writing—review & editing)

Financial support: This study was supported by institutional funding received from the Flinders Foundation, Clinical Genomics Pty Ltd, and the Medical Research Future Funds (application 2009066). The contents of the published material are the responsibility of the authors and do not reflect the views of the Commonwealth.
